# Effects of glyphosate herbicide ingestion on kidney function in rats on a balanced diet

**DOI:** 10.1590/2175-8239-JBN-2023-0043en

**Published:** 2023-12-01

**Authors:** Bruno Reis Moreira Nacano, Marcia Bastos Convento, Andréia Silva de Oliveira, Rafaela Castino, Bianca Castino, Clara Versolato Razvickas, Eduardo Bondan, Fernanda Teixeira Borges

**Affiliations:** 1 Universidade Cruzeiro do Sul, Programa de Pós-Graduação Interdisciplinar em Ciências da Saúde, São Paulo, SP, Brazil.; 2 Universidade Federal São Paulo, Departamento de Medicina, São Paulo, SP, Brazil.; 3 Universidade Paulista, Programa de Pós-Graduação em Patologia Ambiental e Experimental, São Paulo, SP, Brazil.

**Keywords:** Glyphosate, Diet, Nephrotoxicity, Oxidative Stress, Renal Insufficiency, Chronic, Glifosato, Dieta, Nefrotoxicidade, Estresse Oxidativo, Insuficiência Renal, Crônica

## Abstract

**Introduction:**

Glyphosate is the most widely used herbicide worldwide and in Brazil. There is currently increasing concern about the effects of glyphosate on human health. The Brazilian Institute for Consumer Protection showed data on the presence of glyphosate in some of Brazil’s most consumed ultra-processed products. Currently, regulations on the upper limit for these residues in ultra-processed foods have yet to be established by the National Health Surveillance, and ultra-processed food consumption is independently associated with an increased risk of incident chronic kidney disease.

**Methods:**

Since an unbalanced diet can interfere with kidney function, this study aims to investigate the effect of daily intake of 5 mg/kg bw glyphosate in conjunction with a balanced diet and the possible impact on renal function in rats. Kidney function, kidney weight, markers of renal injury, and oxidative stress were evaluated.

**Results:**

There was a decrease in kidney weight. The main histopathological alterations in renal tissues were vacuolation in the initial stage and upregulation of the kidney injury marker KIM-1. Renal injury is associated with increased production of reactive oxygen species in mitochondria.

**Conclusion:**

This study showed changes in the kidney of rats exposed to a balanced diet with glyphosate, suggesting a potential risk to human kidney. Presumably, ultra-processed food that contain glyphosate can potentiate this risk. The relevance of these results lies in drawing attention to the need to regulate glyphosate concentration in ultra-processed foods in the future.

## RESUMO

**Introdução:**

O glifosato é o herbicida mais utilizado no mundo e no Brasil. Atualmente, há uma preocupação crescente com os efeitos do glifosato na saúde humana. O Instituto Brasileiro de Defesa do Consumidor apresentou dados sobre a presença de glifosato em alguns dos produtos ultraprocessados mais consumidos no Brasil. Atualmente, as regulamentações sobre o limite máximo desses resíduos em alimentos ultraprocessados ainda não foram estabelecidas pela Vigilância Sanitária Nacional, e o consumo de alimentos ultraprocessados está indepen­dentemente associado a um risco maior de doença renal crônica incidente.

**Métodos:**

Como uma dieta desbalanceada pode interferir na função renal, este estudo tem como objetivo investigar o efeito da ingestão diária de 5 mg/kg pc de glifosato em conjunto com uma dieta equilibrada e o possível impacto na função renal em ratos. Foram avaliados função renal, peso dos rins, marcadores de lesão renal e estresse oxidativo.

**Resultados:**

Houve redução no peso dos rins. As principais alterações histopatológicas nos tecidos renais foram vacuolização no estágio inicial e regulação positiva do marcador de lesão renal KIM-1. A lesão renal está associada à produção aumentada de espécies reativas de oxigênio nas mitocôndrias.

**Conclusão:**

Esse estudo mostrou alterações nos rins de ratos expostos a uma dieta balanceada com glifosato, sugerindo um risco potencial ao rim humano. Presumivelmente, alimentos ultraprocessados que contenham glifosato podem potencializar esse risco. A relevância desses resultados está no fato de chamar a atenção para a necessidade de regulamentar a concentração de glifosato em alimentos ultraprocessados no futuro.

## Introduction

Glyphosate [N-(phosphonomethyl) glycine] is the most widely used herbicide worldwide^1^. In agriculture, it is mainly used in the production of soybean, corn, cotton, and pasture crops. It may also be used as a plant growth regulator that hastens certain grains and legumes harvest when applied as a drying agent. Non-agricultural uses of glyphosate include forestry, house maintenance, and vegetation control on industrial lands and transportation routs (such as train tracks and highways)^1^. Glyphosate is the most commonly used pesticide marketed in Brazil and accounts for 31.45% of the market^2^.

After exposure, tissue concentrations of glyphosate are highest in the kidneys, followed by spleen, fat, and liver^4^. It is eliminated in the feces (60–70%), and the remaining 20 to 30% is rapidly eliminated in the urine after 48 h the, and after seven days, it is completely excreted from the body^4^.

There is currently increasing concern about the effects of glyphosate on human health, which have been explored in several studies^5,6,7^ to show that glyphosate and glyphosate-based herbicides disrupt the estrogen pathway, impair certain cerebral functions, exhibit cytotoxic and genotoxic effects, increase oxidative stress, cause inflammation, affect lymphocyte functions, and allegedly correlate with some cancers and with chronic kidney disease (CKD) of unknown etiology (CKDu).

The National Health Surveillance Agency (ANVISA) of Brazil provides the following toxicological reference values of glyphosate that are safe for humans: (1) an acceptable daily intake of 0.5 mg/kg body weight (bw); (2) an acute reference dose of 0.5 mg/kg body weight; and (3) an acceptable operator exposure of 0.1 mg/kg body weight^8^.

In 2021 and 2022, the Brazilian Institute for Consumer Protection published data on a novel study in Brazil entitled “There is Poison in This Package (Volume 1 and 2)”. The publication included data on the presence and identification of pesticide residues in some of Brazil’s most consumed ultra-processed products^9^. The study analyzed 24 products divided into eight meat and milk derivative categories. The results showed that 14 of the 24 tested products contained pesticides. Glyphosate and its metabolites were the most common compounds, accounting for 9 of the 24 products analyzed.

Nilson et al.^10^ showed that the consumption of ultra-processed foods was correlated with 57,000 premature deaths in Brazil in 2019, accounting for 10.5% of all preventable deaths that year based on data extracted from DataSUS in Brazil^11^. The problematic question now is whether the presence of glyphosate in ultra-processed products can exacerbate these numbers.

Changes in food matrix (a soft texture that requires less chewing), energy density, and the use of additives in the industrial process amplify sensory properties that increase the eating rate and delay satiety signaling. These changes modulate overconsumption^12^, which may lead to a daily intake of glyphosate at a higher dose than that recommended by ANVISA^8^. Currently, there are no regulations on the upper limit for these residues in ultra-processed foods; ANVISA monitors these substances in natural foods only^9^.

As previously reported in the literature, the Brazilian Ministry of Health also warns that ultra-processed food contains high levels of sugars, fats, and sodium and is linked to the development of chronic diseases^13^. Unbalanced diets are strongly associated with kidney diseases^14^, and we have previously demonstrated the deleterious effects of a high-fat/high-fructose diet on kidney function in rats^15^.

Since an unbalanced diet can interfere with kidney function, this study aimed to investigate the effect of glyphosate ingestion 10-fold above the safe dose reported by ANVISA and the possible impact on renal function in rats on a balanced diet.

## Methods

### Experimental Design

Male Wistar rats weighing 250–300 grams (g) at 30 days were housed in individual boxes with wood shavings, and maintained at 22–24°C, 10% relative humidity, and an alternating 12/12 h light/dark cycle. The balanced diet for the groups was as follows: 20% protein, 61% carbohydrates, 17% lipids, and 10% sucrose (Nuvilab, Colombo, Brazil) and water *ad libitum*. The *in vivo* experimental design was conducted in accordance with Brazilian guidelines^16^ and was approved by the ethics committee (Research Ethics Committee 13667748/2019).

After acclimation for seven days, the rats were randomly assigned to the control group (n = 6) or glyphosate group (n = 6). Rats were treated daily for 25 days with either vehicle (PBS; the control group) or glyphosate (5 mg/kg [Sigma Aldrich, MO, USA]) by oral gavage. The rats were placed in metabolic cages for 24 hours (h) for urine collection, and blood samples were taken from the lateral tail vein.

The rats were euthanized 30 days after the beginning of the experimental protocol through an intraperitoneal injection of a toxic dose of 10 mg/kg of xylazine (Agribrands do Brasil, São Paulo, Brazil) and 90 mg/kg of ketamine (Agribrands do Brasil) and both kidneys were removed for histology and weight analysis.

Body weight was monitored to carefully characte­rize weight gain. The absolute kidney weight and relative kidney weight/body weight were measured with an AD-5000 balance (Marte Científica Ltda, SP, Brazil), and the results are expressed in grams.

### In Vivo Biochemical Analysis

Plasma and urine levels of urea and creatinine were spectrophotometrically assayed according to standard procedures using commercially available diagnostic kits (Labtest Diagnostica, Lagoa Santa, Brazil). The results are expressed as mg/mL. Creatinine clearance was calculated according to the equation: (urine creatinine concentration × urine volume) / (plasma creatinine concentration × 1440), and the results are expressed as mL/min. Urinary protein was established using a colorimetric method based on pyrogallol red-molybdate^17^, and the results are expressed as mg/mL of urinary protein/creatinine and mg/24 h.

### Kidney Tissue

Kidney tissues were embedded in paraffin, sectioned, and stained with hematoxylin and eosin (Erviegas, São Paulo, Brazil). The histopathological changes were analyzed according to the severity of proximal tubular vacuolization. The kidney sample was classified as follows: 0, normal kidney; 1, mild lesion (0-5%); 2, moderate lesion (5–25%); 3, intermediate lesion (25–75%); and 4, severe lesion (75–100%)^18^.

The paraffin sections were subjected to alcohol and xylene gradient solutions, antigen retrieval, and protein blocking. The sections were first incubated with primary antibodies against kidney injury molecule 1 (KIM-1 [1:200, rabbit IgG, H07H, Sino Biologica, BJ, CH]) overnight at 4°C. Subsequently, the sections were incubated with streptavidin-peroxidase for 30 min (Dako, CA, USA). Microscope images were analyzed using the Leica DFC 310 FX (Leica do Brasil Importação e Comércio Ltda, SP, Brazil) image analysis software. The results are expressed as percentages/stained areas.

### Oxidative Stress

Thiobarbituric acid reactive substances (TBARS) in urine form a red compound, the concentration of which was measured by spectrophotometry at 535 nm^19^. The lipid peroxidation levels are expressed as nmol/mg of urinary creatinine. Urinary peroxides were determined using a ferrous oxidation method for orange xylenol version 2 (FOX-2)^20^. The results are expressed as mL/mg of urinary creatinine.

### Statistical Analysis

Descriptive statistical analyses of the data were perfor­med using the Action Stat software (version 3.3.2) for Windows. Data were initially evaluated using the Shapiro-Wilk normality test. Data with non-normal distribution were then evaluated using the Wilcoxon test, while data with normal distribution were compared using the Student’s t-test. The results are reported as the mean ± standard error of the mean. The statistical significance level was set at 5% (p ≤ 0.05).

## Results

**Figure 1.**
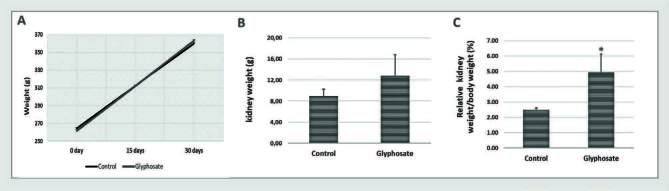
Physiological parameters. The rats were fed a balanced diet (Control) or a balanced diet and glyphosate daily (5mg/kg bw [glyphosate]) for 30 days. **A.** Body weight. **B.** Absolute kidney weight. **C.** Relative kidney weight. The significance level for a null hypothesis was set at 5% (p ≤ 0.05). *Compared to the Control group.

The final body weights of the rats in the experimental groups were similar (Control: 352.51 ± 7.24 g; Glyphosate: 355.50 ± 14.46 g). Figure 1A shows a tendency for a decrease in absolute kidney weight (Control: 1.64 ± 0.07 g; Glyphosate: 1.43 ± 0.08 g) (Figure 1B) and a significant reduction in relative kidney weight/body weight (Control: 0.005 ± 0.000; Glyphosate: 0.004 ± 0.000) in rats exposed to glyphosate compared to the control group (Figure 1C).

**Figure 2.**
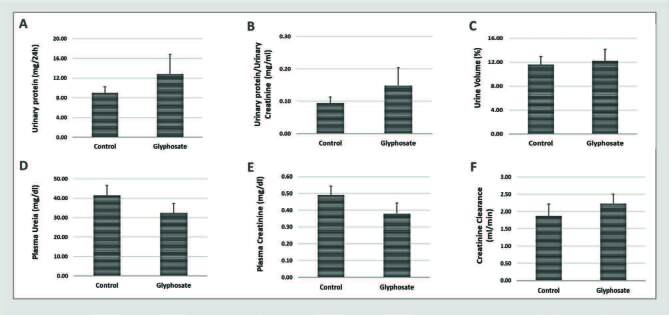
Evaluation of renal function. The rats were fed a balanced diet (Control) or a balanced diet and glyphosate daily (5mg/kg bw [glyphosate]) for 30 days. **A.** Urinary protein. **B.** Urinary protein/urinary creatinine. **C.** Urine volume. **D.** Plasma urea. **E.** Plasma creatinine. **F.** Creatinine clearance. The significance level for a null hypothesis was set at 5% (p ≤ 0.05). *Compared to the Control group.

Figure 2A–F shows the results of kidney function evaluation. There were no differences in plasma urea (Control: 41.50 ± 5.20; Glyphosate: 35.60 ± 5.00), plasma creatinine levels (Control: 0.49 ± 0.05; Glyphosate: 0.38 ± 0.06), creatinine clearance (Control: 1.87 ± 0.35; Glyphosate: 2.34 ± 0.08), urine volume (Control: 11.57 ± 1.38; Glyphosate: 12.25 ± 1.92), and a tendency for a higher proteinuria (Control: 0.09 ± 0.02; Glyphosate: 0.18 ± 0.04) in rats exposed to glyphosate compared to rats in the control group at 30 days.

**Figure 3.**
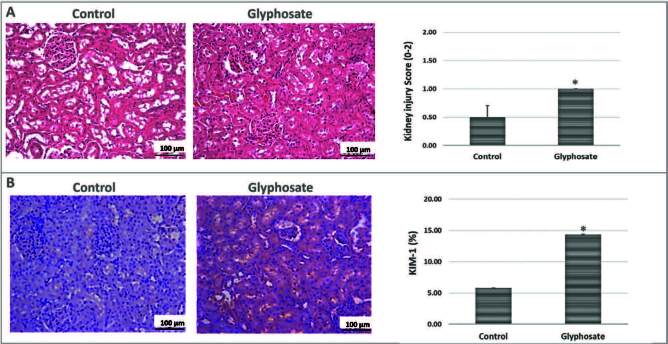
Evaluations in the renal tissue. The rats were fed a balanced diet (Control) or a balanced diet and glyphosate daily (5mg/kg bw [glyphosate]) for 30 days. **A.** Histopathological analysis of kidneys paraffin sections stained with hematoxylin and eosin and their quantitative analyses of kidney injury score (1–4). **B.** Light microscopy of kidney injury molecule-1 (KIM-1) and its quantitative analyses of stained kidney sections. The significance level for a null hypothesis was set at 5% (p ≤ 0.05). *Compared to the Control group.

The histopathological changes were analyzed regarding the severity of proximal tubular vacuoliza­tion at 30 days in Figure 3A. Vacuolation in tubular cells at an initial stage were found in rats exposed to glyphosate compared to the control group (Control: 0.50 ± 0.20; Glyphosate: 1.00 ± 0.00). Figure 3B shows significantly higher KIM-1 levels in rats exposed to glyphosate than in the control group rats (Control: 5.82 ± 0.01; Glyphosate: 14.34 ± 0.00).

**Figure 4.**
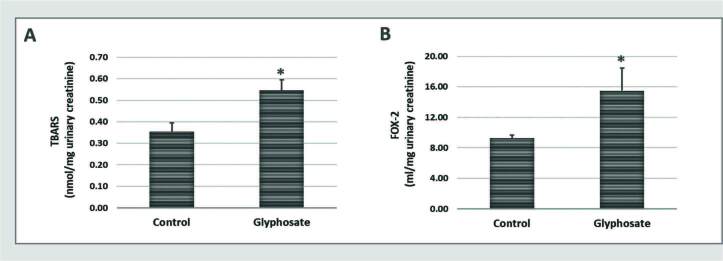
Reactive oxygen species (ROS) in urine. The rats were fed a balanced diet (Control) or a balanced diet and glyphosate daily (5mg/kg bw [glyphosate]) for 30 days. **A.** Quantitative analyses of thiobarbituric reactive substances (TBARS). **B.** Urinary peroxides (FOX-2). The significance level for a null hypothesis was set at 5% (p ≤ 0.05). *Compared to the Control group.

Figure 4 shows the urinary levels of TBARS (A) and FOX-2 (B). Analyses of TBARS (Control: 0.35 ± 0.04; Glyphosate: 0.55 ± 0.05) and FOX-2 (Control: 9.28 ± 0.37; Glyphosate: 15.50 ± 2.97) levels showed that lipid peroxidation was increased in rats exposed to glyphosate compared to the control group.

## Discussion

The majority of epidemiological studies on glyphosate are case-control and cohort studies that examined potential associations of glyphosate exposure in humans; there is no information on the health effects on humans exposed to different doses of glyphosate^21^. The most reliable dose-response data come from studies in animals administered glyphosate orally^21^.

The Acceptable Daily Intake (ADI) was defined by ANVISA based on the available long-term studies and international discussions. The “*no observed adverse effect level* (NOAEL)” used to calculate the ADI was the same found in developmental studies in rabbits (50 mg/kg bw per day). Considering the standard 100-fold safety factor, the new ADI for glyphosate is 0.5 mg/kg bw^8^.

Roman et al.^22^ used dosages of 15, 75, and 150 mg/kg diluted in drinking water applied once a day by gavage for 20 to 25 days and found homogeneity between renal tubules and cytoplasmic vacuolization in the tubule in microscopical analyses of the kidney of experimental animals. However, these doses are not found in foods, and the study investigated the effect of glyphosate ingestion ten-fold higher than the safe dose reported by ANVISA^8^ and revealed the impact of combining glyphosate and a balanced diet on renal function in rats.

For repeated-dose toxicity studies in animals, the American Society of Toxicological Pathology^23^ and the United States Environmental Protection Agency guidelines^24^ recommend regular weighing and histopathological examinations of the kidneys to detect chemically induced renal toxicity. Nephrotoxins act by causing either an increase or a decrease in kidney weight^23-25^. The results of this study suggest that glyphosate toxicity decreases kidney weight.

Tubular vacuolization is observed in toxic injuries, xenobiotic-induced renal injuries, ischemic conditions, and other renal diseases^26^. The molecular mechanism is unclear but likely reflects changes in the concentration of certain circulating compounds caused indirectly or directly by chronic suppression of cytochrome P450 reductase expression^27^. Glyphosate induces the suppression of cytochrome p450^28^. Larsen et al.^29^ estimated that glyphosate causes a ~50% reduction in cytochrome P450 levels in rats. Our data indicated tubular vacuolization in the initial stages of glyphosate exposure in the rats.

After extensive research in the field, the Food and Drug Administration and the European Medicines Agency approved biomarkers for nephrotoxicity detection including kidney injury molecule-1 (KIM-1), which is a proximal tubule transmembrane protein^30^. KIM-1 is used to identify and monitor substance-induced kidney injury^31^ and many other causes of kidney injury. This biomarker was approved as a nephrotoxic biomarker by the Food and Drug Administration over a decade ago^30^. Our results confirmed glyphosate (5mg/kg bw)-induced KIM-1 expression in the kidneys of glyphosate-exposed rats.

After proximal tubular injury, matrix metalloproteinases cleave the extracellular domain of KIM-1, which appears in the urine of humans^32^. It has been recognized as a sensitive, early urinary biomarker specific to kidney injury in rodents and humans^33^. The presence of elevated circulating levels of KIM-1 in blood has been associated with acute and chronic kidney damage^34^.

Oxidative stress also stimulates KIM-1 expression. The signaling transducer and activator of the transcription 3 (STAT3) pathway are linked to oxidative stress^31^ since nuclear STAT3 binds to the KIM-1 promoter, stimulating their expression^35^. Exposure to glyphosate positively impacts oxidative stress and increases lipid peroxidation by 130%^36^. Oxidative damage occurs when reactive oxygen species attack the double bonds of unsaturated fat in cell membranes and produce various lipid peroxidation products, such as TBARS and FOX-2. Herein, rats exposed to glyphosate for 30 days showed increased reactive oxygen species demonstrated by increased TBARS and FOX-2.

This increase in reactive oxygen species results in kidney cell damage through the suppression of cellu­lar respiration, adenosine triphosphate production, cytochrome-c release from the mitochondrial membr­ane, lipid peroxidation, cell membrane destabilization, resulting in necrosis and ultimately cell death^37^.

The change in the kidney of rats subjected to a balanced diet with glyphosate indicate a potential risk to renal function in humans. Ultra-processed food is independently associated with an increased risk of CKD^14,15^, and presumably, ultra-processed food containing glyphosate can potentiate this risk. It is estimated that 1.5% of the Brazilian population has already developed CKD^38^. Therefore, a strategy for risk prevention is necessary.

Adult patients are diagnosed with CKD if it has been present for three months or more^38,39^, if they present a glomerular filtration rate (GFR) below 60 mL/min/1.73 m^2^ or GFR above 60 ml/min/1.73 m^2^ but with evidence of injury to the kidney structure. Albuminuria, imaging renal changes, persistent hydroelectrolytic disorders, hematuria/leukocyturia, histological changes in kidney biopsy, and previous kidney transplantation are all indicators of renal injury^38,39^. The presence of more than 30 mg of albumin in the 24-hour urine or more than 30 mg/g of albumin defines albuminuria^38,39^.

Our data showed that the change in kidney weight was related to glyphosate ingestion. The main histopathological alterations in kidney tissues were vacuolation in the initial stage with upregulation of the kidney injury marker KIM-1, considering that oxidative stress also stimulates KIM-1 expression.

In conclusion, these findings suggest an increased risk of progressive loss of kidney function over time. It is necessary to evaluate the long-term effects of glyphosate ingestion and how constant injury may contribute to the development of chronic kidney disease. Thus, the relevance of these results is that they draw attention to the need to regulate the concentration of glyphosate in ultra-processed foods in the future, prioritize consumer safety, and conduct a thorough dietary risk assessment. The dose makes the poison.

## Data Availability

All data generated or analyzed during this study are included in this article.

## Abbreviations

ADI: – Acceptable daily intake
CKD: – Chronic kidney disease
ANVISA: – National Health Surveillance Agency
IDEC: – Brazilian Institute for Consumer Protection
TBARS: – Thiobarbituric acid reactive substances
KIM-1: – Kidney injury molecule
FOX-2: – Orange xylenol version 2
STAT3: – Signaling transducer and activator of transcription 3
GFR: – Glomerular filtration rate
NOAEL: – No observed adverse effect level
